# The trans-membrane domain of Bcl-2α, but not its hydrophobic cleft, is a critical determinant for efficient IP_3_ receptor inhibition

**DOI:** 10.18632/oncotarget.11005

**Published:** 2016-08-02

**Authors:** Hristina Ivanova, Abigael Ritaine, Larry Wagner, Tomas Luyten, George Shapovalov, Kirsten Welkenhuyzen, Bruno Seitaj, Giovanni Monaco, Humbert De Smedt, Natalia Prevarskaya, David I. Yule, Jan B. Parys, Geert Bultynck

**Affiliations:** ^1^ Katholieke Universiteit Leuven, Laboratory of Molecular and Cellular Signaling, Department of Cellular and Molecular Medicine and Leuven Cancer Institute (LKI), BE-3000 Leuven, Belgium; ^2^ Inserm U-1003, Equipe Labellisée par la Ligue Nationale Contre le Cancer et LABEX (Laboratoire d'excellence), Université Lille1, 59655 Villeneuve d'Ascq, France; ^3^ University of Rochester Medical Center School of Medicine and Dentistry, Rochester, NY 14642, USA

**Keywords:** calcium signaling, anti-apoptotic Bcl-2, trans-membrane domain, hydrophobic cleft, IP_3_ receptor

## Abstract

The anti-apoptotic Bcl-2 protein is emerging as an efficient inhibitor of IP_3_R function, contributing to its oncogenic properties. Yet, the underlying molecular mechanisms remain not fully understood. Using mutations or pharmacological inhibition to antagonize Bcl-2's hydrophobic cleft, we excluded this functional domain as responsible for Bcl-2-mediated IP_3_Rs inhibition. In contrast, the deletion of the C-terminus, containing the trans-membrane domain, which is only present in Bcl-2α, but not in Bcl-2β, led to impaired inhibition of IP_3_R-mediated Ca^2+^ release and staurosporine-induced apoptosis. Strikingly, the trans-membrane domain was sufficient for IP_3_R binding and inhibition. We therefore propose a novel model, in which the Bcl-2's C-terminus serves as a functional anchor, which beyond mere ER-membrane targeting, underlies efficient IP_3_R inhibition by (i) positioning the BH4 domain in the close proximity of its binding site on IP_3_R, thus facilitating their interaction; (ii) inhibiting IP_3_R-channel openings through a direct interaction with the C-terminal region of the channel downstream of the channel-pore. Finally, since the hydrophobic cleft of Bcl-2 was not involved in IP_3_R suppression, our findings indicate that ABT-199 does not interfere with IP_3_R regulation by Bcl-2 and its mechanism of action as a cell-death therapeutic in cancer cells likely does not involve Ca^2+^ signaling.

## INTRODUCTION

**Figure 1:**
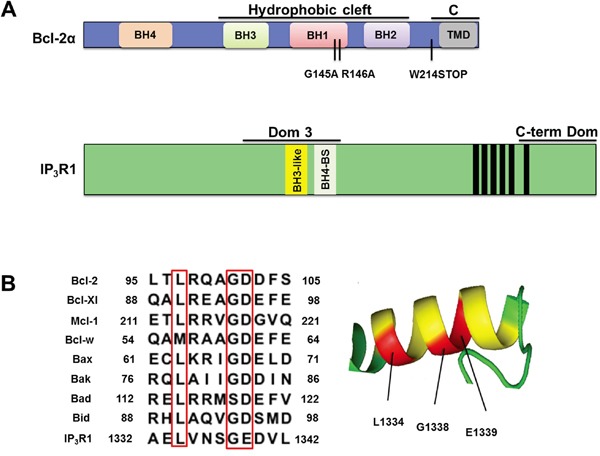
The Dom 3 of IP_3_R1 contains a BH3 motif **A.** Linear representation of Bcl-2 and IP_3_R1. Bcl-2α is depicted in blue with its BH domains and the trans-membrane domain (TMD). The two functional domains of interest, the hydrophobic cleft formed by BH3-BH1-BH2 domains and the C-terminus (C), are indicated with black lines. The C-terminal region, containing the TMD, is present in Bcl-2α, but not in Bcl-2β. The G145 and R146 residues, located in the BH1 domain were mutated to yield Bcl-2^GR/AA^. Bcl-2 was truncated at W214 residue to yield Bcl-2^ΔC^, which correlates with Bcl-2β. A schematic representation of IP_3_R1 is depicted in green. The Bcl-2-binding fragments of IP_3_R used in this study, the domain 3 (Dom 3) and the C-terminal domain containing the last TMD (C-term Dom), are indicated with black lines and the six TMDs are shown as black bars. The exact BH4-binding site in the Dom 3 is represented in light grey (BH4-BS). The BH3-like motif in the Dom 3 is represented in yellow. **B.** The Dom 3 of IP_3_R1 contains a BH3 motif. Left: Sequence alignment between the BH3 domains of Bcl-2 family members and the Dom 3 of IP_3_R1 reveals the presence of the conserved residues (LxxxGD/E, pointed in red), required for a typical BH3 motif (46). Right: A secondary structure prediction of the putative BH3 motif of IP_3_R1 present in the Dom 3 sequence, predicted by the I-TASSER web server and drawn using PyMol. The predicted BH3 motif within the Dom 3 is depicted in yellow and the conserved residues in red.

A hallmark of cancer cells is their ability to prolong cell survival by avoiding apoptosis. The family of B-cell lymphoma-2 (Bcl-2) proteins is a critical regulator of this process [1–5]. It consists of anti-apoptotic members, including Bcl-2 [6] and Bcl-Xl [7] and pro-apoptotic members like Bax [8]. All the members of the family share at least one of the four conserved α-helical motifs, known as Bcl-2 homology (BH1-4) domains [1, 9]. Many of these proteins exist in more than one isoforms [7, 10–13] and Bcl-2 is not an exception. Two isoforms of Bcl-2, resulting from alternative splicing, were described: Bcl-2α and Bcl-2β [14]. Most of the work until now has been done with Bcl-2α, which is the long isoform and which in addition to the four BH domains contains a C-terminal extension with a putative trans-membrane domain (TMD) (Figure 1A). In contrast, Bcl-2β has a much shorter C-terminus and lacks a TMD [14, 15]. While Bcl-2β is mostly detected in cytosolic fractions, the TMD and a short preceding sequence target Bcl-2α to a variety of intracellular membranes including mitochondrial, endoplasmic reticulum (ER) and nuclear membranes [16–18]. Bcl-2α is the more abundant isoform in both healthy and cancer cells and it remains dominant in cancer cells up-regulating Bcl-2 protein [14, 19]. In virtually all studies published to this date, Bcl-2 refers to Bcl-2α. The anti-apoptotic function of Bcl-2 oncogene was first characterized at the level of the mitochondria, particularly at the outer mitochondrial membrane, where it inhibits Bax/Bak–mediated apoptosis. The mechanism involves a BH3-dependent interaction, where the hydrophobic cleft of Bcl-2 formed by the BH3-BH1-BH2 domains sequesters the BH3 domain of the pro-apoptotic members. This prevents Bax/Bak activation and oligomerization and inhibits the consequent mitochondrial permeabilization and cell death [2, 3, 20, 21].

Ca^2+^ signaling is another important modulator in cell-fate decisions, which can serve as a survivor factor promoting cell proliferation, but also as a cell-death inducer [22–25]. Bcl-2 was shown to execute its pro-survival function not only *via* direct inhibition of pro-apoptotic proteins but also *via* suppression of pro-apoptotic Ca^2+^ signals. This occurs by direct interaction with inositol 1,4,5-trisphosphate (IP_3_) receptors (IP_3_Rs) [26–29], the main intracellular Ca^2+^ release channels, located at the ER [30–34]. IP_3_R inhibition by Bcl-2 appears to be an important mechanism that contributes to the oncogenic properties of Bcl-2. Many cancer cells, including leukemia, lymphoma and lung cancer cells, are addicted to IP_3_R/Bcl-2-complex formation for their survival, since tools that disrupt this complex trigger cell death [35–37]. Over the last years, important insights in the regulation of IP_3_Rs by Bcl-2 (i.e. Bcl-2α) at the molecular level have been obtained. The suppression of IP_3_R-mediated Ca^2+^ release by Bcl-2 was attributed to the interaction of the BH4 domain of Bcl-2 with a 20 amino acid region (a.a. 1389-1408) located in the central modulatory domain, more particularly in the Domain 3 (Dom 3) (a.a. 923-1581) of IP_3_R [38, 39]. Previous studies, which exploited synthetic peptides covering the BH4 domain of Bcl-2 (BH4-Bcl-2), revealed that this domain is necessary and sufficient to bind to IP_3_R and to suppress its activity [26, 27, 39, 40]. Nevertheless, the relatively low affinity of inhibition by the BH4 domain (measured *in vitro* IC_50_=30μM) [27, 39] cannot explain the potent inhibitory effect of Bcl-2 full-length protein in physiological conditions. This Achilles' heel of the model suggests that additional domains in Bcl-2 could be responsible for an efficient *in cellulo* inhibition of IP_3_R. Interestingly, the C-terminal domain, containing the last 6^th^ TMD of the IP_3_R (C-term Dom, a.a. 2512-2749), which is in close proximity of the channel pore is also targeted by Bcl-2 [41, 42], but the mechanism and significance of this interaction are not completely solved. The same C-term Dom of IP_3_R also appeared to be responsible for interaction with other members of the family: Bcl-Xl and Mcl-1 [41].

Here, we aimed to identify the molecular determinants in Bcl-2α responsible for its interaction with the C-term Dom of IP_3_R and to assess their functional impact on Bcl-2α-mediated inhibition of the channel. We especially focused on two important functional domains in Bcl-2α, i.e. the hydrophobic cleft, involved in BH3-dependent interactions and the C-terminal region, containing the TMD, involved in hydrophobic interactions within the membrane environment (Figure 1A). Using genetic and pharmacological approaches, we could however exclude the hydrophobic cleft as a major player in the formation of the Bcl-2α/IP_3_R complex. In contrast, we found that Bcl-2α binding to the C-term Dom of IP_3_R1 depends on the presence of the C-terminus of Bcl-2α. This region of Bcl-2α is required for efficient inhibition of IP_3_Rs in a cellular context and for inhibition of staurosporine (STS) – induced apoptosis. Furthermore, we demonstrated a direct interaction between a peptide corresponding to the TMD of Bcl-2α (TMD-Bcl-2) and the purified C-terminal fragment of IP_3_R1. The TMD-Bcl-2 was able to suppress IP_3_-induced Ca^2+^ release (IICR) when applied at high concentrations. These results suggest that the C-terminal region, and particularly the TMD, of Bcl-2α not only serves as an anchor for tethering Bcl-2α in the membranes, but is also an important functional regulator of IP_3_R activity. Since the TMD is only present in Bcl-2α, but not in Bcl-2β, this study is the first one hinting towards important functional difference between the two isoforms with respect to Ca^2+^-signaling regulation.

## RESULTS

### Despite the presence of BH3-domain features in the IP_3_R sequence, the hydrophobic cleft of Bcl-2α is dispensable for interaction with the receptor

We performed a sequence alignment of the BH3 domains of different Bcl-2 proteins with the fragment of the central modulatory domain of IP_3_R1 (Dom 3), shown in previous studies to bind Bcl-2 [27, 38, 42]. This analysis revealed the presence of a BH3 motif (a.a. 1332-1342) upstream of the previously described region in Dom 3 of IP_3_R targeted by the BH4 domain of Bcl-2 (a.a. 1389-1408) (Figure 1A) [43]. Figure 1B depicts the presence of the conserved LxxxGD/E motif [44] in the Dom 3 of IP_3_R and the α-helical secondary structure of this motif as predicted by I-TASSER web server. To determine whether a BH3-dependent mechanism plays a direct role in the interaction between Bcl-2α and IP_3_R we used two different approaches to antagonize the hydrophobic cleft of Bcl-2α, genetic manipulation and pharmacological inhibition. The genetic approach is based on mutations in the BH1 domain (replacement of G145R146 by AA yielding Bcl-2^GR/AA^) (Figure 1A), which lead to disruption of the binding between Bcl-2α and Bax [45–47]. The second approach is based on the use of pharmacological inhibitors like the BH3-mimetic compounds [48, 49], designed to occupy the hydrophobic cleft, thereby disrupting interactions between BH3 domain-containing proteins and anti-apoptotic Bcl-2 proteins [48, 49]. Here, we applied ABT-199, a selective Bcl-2 inhibitor which does not target Bcl-Xl [50].

**Figure 2:**
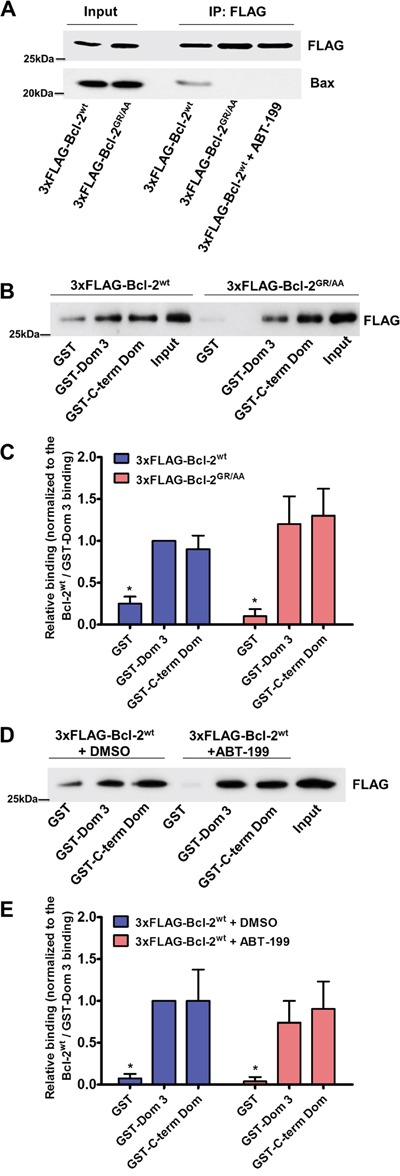
Bcl-2GR/AA and Bcl-2wt exposed to ABT-199 fail to bind pro-apoptotic Bax, but remain capable of binding the Dom 3 and the C-term Dom of IP_3_R **A.** Representative FLAG-co-immunoprecipitation experiment for detection of the 3xFLAG-Bcl-2/Bax interaction is shown. Overexpressed 3xFLAG-Bcl-2^wt^ (in absence and presence of 3 μM ABT-199) or 3xFLAG-Bcl-2^GR/AA^ was immunoprecipitated from COS-1 cell lysates by anti-FLAG-loaded agarose beads. The immunoreactive blots were stained with antibody against FLAG and Bax. 0.1 μg and 0.5 μg of total COS-1 lysates were used as input for the 3xFLAG-proteins and Bax respectively. The experiments were performed 3 times utilizing each time independently transfected cells and freshly prepared lysates. **B.** Representative GST-pull down experiments with COS-1 cell lysates for comparing the binding of overexpressed 3xFLAG-Bcl-2^wt^ and 3xFLAG-Bcl-2^GR/AA^ to the GST-Dom 3 and GST-C-term Dom are shown. The samples were analyzed *via* Western blot and stained with anti-FLAG antibody. The binding to the GST was used as a negative control. 0.1 μg of total COS-1 lysates was used as input. **C.** The immunoreactive bands from 3 independent experiments, utilizing each time independently transfected cells and freshly prepared lysates, were quantified and normalized to the binding of 3xFLAG-Bcl-2^wt^ to GST-Dom 3, which was set as 1. The data are plotted as mean ± S.E.M. **D.** Representative GST-pull down experiments with COS-1 cell lysates for comparing the binding of overexpressed 3xFLAG-Bcl-2^wt^ in absence and presence of 3 μM ABT-199 to the GST-Dom 3 and GST-C-term Dom are shown. The samples were analyzed *via* Western blot and stained with anti-FLAG antibody. The binding to the GST was used as a negative control. 0.1 μg of total COS-1 lysates was used as input. **E.** The immunoreactive bands from 3 independent experiments, utilizing each time independently transfected cells and freshly prepared lysates, were quantified and normalized to the binding of 3xFLAG-Bcl-2^wt^ to GST-Dom 3, which was set as 1. The data are plotted as mean ± S.E.M.

First, we validated that both, the GR/AA mutation or the incubation with ABT-199 (3 μM), prevent Bcl-2α binding to Bax in co-immunoprecipitation experiments. The concentration of ABT-199 that we used in the experiments is well above the documented subnanomolar affinity of this compound for Bcl-2 (Ki < 0.01 nM) [50], thus maximizing the potential effect of ABT-199 on Bcl-2/IP_3_R interaction. 3xFLAG-tagged proteins (3xFLAG-Bcl-2^wt^ in presence and absence of ABT-199 or 3xFLAG-Bcl-2^GR/AA^) were overexpressed in COS-1 cells and immunoprecipitated from the cell lysates using anti-FLAG-loaded agarose beads. Immunoblots were stained for FLAG and Bax (Figure 2A).

Next we performed two different sets of GST pull-down experiments, using the two purified IP_3_R domains targeted by Bcl-2, GST-Dom 3 (a.a. 923-1581) and GST-C-term Dom (a.a. 2512-2749). To compare the binding properties of the wild-type Bcl-2 protein *versus* the mutant for these IP_3_R fragments we overexpressed 3xFLAG-Bcl-2^wt^ or 3xFLAG-Bcl-2^GR/AA^ in COS-1 cells. The binding of 3xFLAG-Bcl-2^wt^ to GST-Dom 3 was used as reference and all binding values were normalized to this control. Our results show that 3xFLAG-Bcl-2^GR/AA^ remained fully capable of binding to both GST-Dom 3 and GST-C-term Dom to a similar extent as 3xFLAG-Bcl-2^wt^ (Figure 2B).

As a second approach, we examined the interaction between Bcl-2^wt^ and the two GST-fused domains of IP_3_R in presence or absence of the BH3-mimetic compound ABT-199 (3 μM). Incubation with ABT-199 did not significantly affect the binding of 3xFLAG-Bcl-2^wt^ to the GST-Dom 3, nor to the GST-C-term Dom (Figure 2C).

Taken together, these results suggest that the hydrophobic cleft of Bcl-2 is dispensable for its interaction with IP_3_R.

### The hydrophobic cleft of Bcl-2α does not contribute to the inhibitory effect on IP_3_Rs

**Figure 3:**
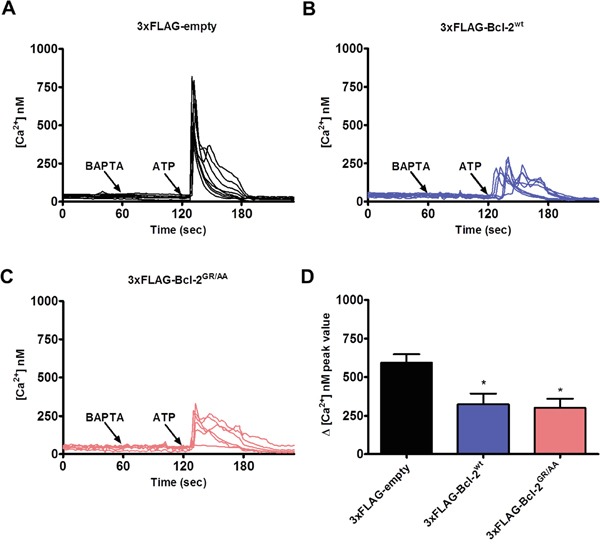
Bcl-2GR/AA remains capable of inhibiting agonist-induced Ca2+ release **A-C.** Intracellular Ca^2+^ release in response to 0.5 μM ATP was followed in the mCherry-positive Fura-2-AM loaded COS-1 cells overexpressing 3xFLAG-empty vector (A), 3xFLAG-Bcl-2^wt^ (B) or 3xFLAG-Bcl-2^GR/AA^ (C). The free extracellular Ca^2+^ was buffered by addition of 3 mM BAPTA. The obtained Fura-2 fluorescence signals (F340/F380) were calibrated and representative traces are plotted as [Ca^2+^]. **D.** Quantitative analysis of the amplitude of the ATP-induced Ca^2+^ signals from at least 3 independent experiments (n > 80 cells) is plotted as mean ± S.E.M.

**Figure 4:**
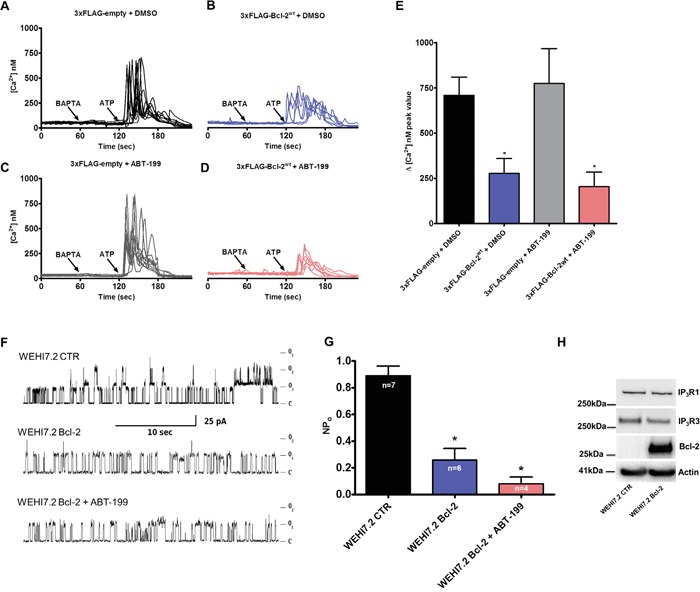
ABT-199 does not impact Bcl-2's ability to suppress IP_3_R activity in single-cell measurements and in patch-clamp single-channel recordings **A-D.** Intracellular Ca^2+^ release in response to 0.5 μM ATP was followed in the mCherry-positive Fura-2-AM loaded COS-1 cells overexpressing 3xFLAG-empty vector (A, C) or 3xFLAG-Bcl-2^wt^ (B, D) in absence (A, B) or presence of 3 μM ABT-199 (C, D). The free extracellular Ca^2+^ was buffered by addition of 3 mM BAPTA. The obtained Fura-2 fluorescence signals (F340/F380) were calibrated and representative traces are plotted as [Ca^2+^]. **E.** Quantitative analysis of the amplitude of ATP-induced Ca^2+^ signals from at least 3 independent experiments (n > 80 cells) is plotted as mean ± S.E.M. **F.** Representative IP_3_R currents in ER-containing membrane fractions from control (WEHI7.2 CTR) (top) and Bcl-2-expressing WEHI7.2 cells without (middle) or with (bottom) application of 1 μM ABT-199. The IP_3_R activity was triggered by 5 μM IP_3_ and 1 μM Ca^2+^ **G.** The mean levels of IP_3_R activity (NP_o_) under these conditions are summarized and the data are plotted as mean ± S.E.M. The total number of recordings for each condition is indicated within every bar. **H.** Western blot analysis of the expression levels of Bcl-2, IP_3_R1 and IP_3_R3 in WEHI7.2 CTR and WEHI7.2 Bcl-2 cells. 5 μg of total lysate was loaded and the immunoreactive bands were stained against Bcl-2, IP_3_R1, IP_3_R3 and actin.

Bcl-2 overexpression results in dampened IP_3_R-mediated Ca^2+^ release in intact cells [27, 29, 39, 51], but whether this effect is mediated through the hydrophobic cleft of Bcl-2 is not known. To address this question, we monitored the change in cytosolic Ca^2+^ levels in response to an IP_3_R agonist, ATP, using the ratiometric fluorescent Ca^2+^ dye Fura-2-AM. Similarly to the GST-pull down experiments, we used the mutation (Bcl-2^GR/AA^) or ABT-199 to antagonize the hydrophobic cleft of Bcl-2α. Intact COS-1 cells overexpressing 1) 3xFLAG-empty vector, 3xFLAG-Bcl-2^wt^ or 3xFLAG-Bcl-2^GR/AA^ and 2) 3xFLAG-empty vector or 3xFLAG-Bcl-2^wt^ in presence or absence of ABT-199 (3 μM), and co-transfected with mCherry plasmid were exposed to ATP (0.5 μM). The proper expression of the 3xFLAG-proteins in the COS-1 cells was assessed via Western blotting using anti-FLAG antibody (Supplementary Figure S1A and S1B). Importantly, the expression levels of 3xFLAG-Bcl-2^wt^ and 3xFLAG-Bcl-2^GR/AA^ proteins were similar, although 3xFLAG-Bcl-2^GR/AA^ tended to be expressed at slightly higher levels. In addition, only cells with similar intensity of mCherry, thus similar levels of 3xFLAG-proteins were subjected to measurement. To chelate the free extracellular Ca^2+^, the experiments were performed in the presence of BAPTA (3 mM), an extracellular Ca^2+^ buffer, ensuring that the ATP-induced [Ca^2+^] rise is only due to Ca^2+^ release from intracellular stores. The ER Ca^2+^-store content was also assessed by applying thapsigargin (Tg, 1 μM), an irreversible SERCA inhibitor, in the presence of BAPTA (Supplementary Figure S1A and Supplementary Figure S1B). Consistent with our previous studies, overexpression of 3xFLAG-Bcl-2^wt^ inhibited ATP-induced Ca^2+^ release without affecting the ER Ca^2+^-stores content [27]. In line with our GST-pull down experiments, neither the overexpression of 3xFLAG-Bcl-2^GR/AA^ (Figure 3A-3C), nor the presence of ABT-199 (Figure 4A-4D) prevented this effect. The quantitative analysis indicated that 3xFLAG-Bcl-2^wt^, 3xFLAG-Bcl-2^GR/AA^ (Figure 3D) and 3xFLAG-Bcl-2^wt^ in presence of ABT-199 (Figure 4E) were equally potent in inhibiting IP_3_R-mediated Ca^2+^ release.

Finally, to underpin that IP_3_R inhibition by Bcl-2α is not affected by ABT-199, we performed direct IP_3_R single-channel measurements by using patch-clamp recordings on giant unilamellar vesicles (GUVs) prepared from the ER membrane fractions of native WEHI7.2 cells, which do not express any of the Bcl-2 isoforms (WEHI7.2 control) or Bcl-2α-overexpressing WEHI7.2 cells (WEHI7.2 Bcl-2). Figure 4F, 4G presents a comparison of the measured IP_3_R-mediated channel current after application of IP_3_ (5 μM) and Ca^2+^ (1 μM). The results demonstrate a significant inhibition of IP_3_R activity in the presence of Bcl-2α, measured as the open probability (NP_o_). The NP_o_ value of 0.89 ± 0.07 for the WEHI7.2-control cells decreased to 0.26 ± 0.09 for WEHI7.2 Bcl-2 cells. Application of ABT-199 (1 μM) could not alleviate the inhibitory effect of Bcl-2α on IP_3_R single-channel opening (NP_o_ 0.08 ± 0.05).

Collectively, these functional experiments based on independent approaches exclude a major contribution of the hydrophobic cleft of Bcl-2α for inhibiting IP_3_R-mediated Ca^2+^ flux.

### The C-terminal region of Bcl-2α is critical for its interaction with the C-term Dom, but not with the Dom 3 of IP_3_R1

**Figure 5:**
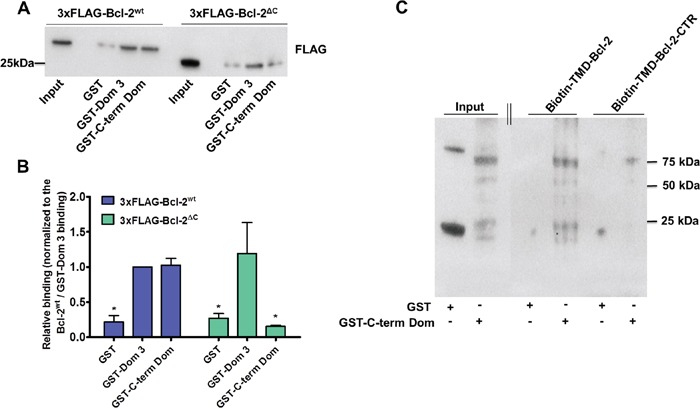
Bcl-2 requires its TMD for binding to the C-term Dom, but not to the Dom 3 of IP_3_R1 **A.** Representative GST-pull down experiments to compare the binding properties of 3xFLAG-Bcl-2^wt^ *versus* 3xFLAG-Bcl-2^ΔC^ overexpressed in COS-1 cells for GST-Dom 3 and GST-C-term Dom are shown. The binding to GST is used as a negative control. 0.1 μg of total COS-1 lysates was used as input. **B.** The immunoreactive bands from 4 independent experiments, utilizing each time independently transfected cells and freshly prepared lysates, were quantified and normalized to the binding of 3xFLAG-Bcl-2^wt^ to GST-Dom 3, which was set as 1. The data are plotted as mean ± S.E.M. **C.** Representative biotin-pull down experiment to study the binding of biotin-TMD-Bcl-2 or biotin-TMD-Bcl-2-CTR peptide to the purified GST or GST-C-term Dom is shown. The immunoblots were stained for GST. The experiment was performed 3 times. 0.2 μg of purified GST and GST-C-term Dom was loaded as input. The double line indicates that two parts of the same immunoblot and exposure time were merged together.

After demonstrating that the hydrophobic cleft of Bcl-2α is not involved in the binding to and inhibition of IP_3_R, we investigated whether the C-terminal region containing the TMD of Bcl-2 could serve as an IP_3_R-interaction domain. We studied the binding of 3xFLAG-Bcl-2 lacking its C-terminal region (3xFLAG-Bcl-2^ΔC^) to purified GST-Dom 3 and GST-C-term Dom using GST-pull-down assays. In these experiments, consistent with our previous results, 3xFLAG-Bcl-2^wt^ bound with equal efficiency both IP_3_R GST-domains [42]. In line with previous data, showing that the BH4 domain of Bcl-2 is sufficient to bind to the Dom 3 [27, 39], 3xFLAG-Bcl-2^ΔC^ remained capable to bind to this domain. Yet, the interaction with GST-C-term Dom was severely impaired (Figure 5A, 5B). These results suggest that while the C-terminal region of Bcl-2α is not crucial for interaction with the Dom 3, it is essential for binding to the C-term Dom of IP_3_R.

### 3xFLAG-Bcl-2^wt^, 3xFLAG-Bcl-2^GR/AA^ and 3xFLAG-Bcl-2^ΔC^ bind to the full-size IP_3_R

3xFLAG-Bcl-2 mutants seem to have differential binding properties for the different IP_3_R domains. However, the performed FLAG-co-immunoprecipitation experiments with lysates from COS-1 cells overexpressing 3xFLAG-empty vector, 3xFLAG-Bcl-2^wt^, 3xFLAG-Bcl-2^GR/AA^ or 3xFLAG-Bcl-2^ΔC^ revealed that the wild type and both mutated proteins are able to interact with the endogenous IP_3_R1 (Supplementary Figure S2). These data are consistent with previous studies showing that the BH4 domain of Bcl-2 is the major determinant for binding to IP_3_Rs [27].

We also compared the binding properties of 3xFLAG-Bcl-2^wt^, 3xFLAG-Bcl-2^GR/AA^ and 3xFLAG-Bcl-2^ΔC^ for endogenous pro-apoptotic Bax. As expected, 3xFLAG-Bcl-2^GR/AA^ failed to interact with Bax. The truncated Bcl-2 displayed equal efficiency for binding Bax as the wild type Bcl-2, confirming that the hydrophobic cleft is the major binding determinant in Bcl-2 interactions with pro-apoptotic proteins (Supplementary Figure S2).

### The TMD of Bcl-2α directly interacts with the C-term Dom of IP_3_R1

As a next step we performed pull-down experiments using neutravidin-coated beads that captured the biotinylated peptides corresponding either to the TMD of Bcl-2 (biotin-TMD-Bcl-2) or to a control version in which several hydrophobic residues were substituted by charged amino acids (biotin-TMD-Bcl-2-CTR) in the presence of either purified parental GST or purified GST-C-term Dom of IP_3_R1. After incubation and washing steps, the resulting pull-down samples were analysed *via* immunoblotting using anti-GST antibody (Figure 5C). This analysis revealed a direct interaction between the GST-C-term Dom of IP_3_R1 and biotin-TMD-Bcl-2.

### The lack of the C-terminus leads to loss of Bcl-2α's ability to suppress IP_3_R-mediated Ca^2+^ release

**Figure 6:**
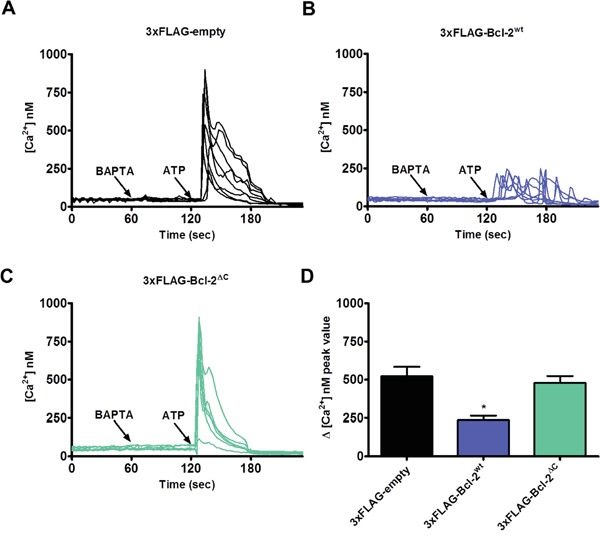
Bcl-2ΔC fails to inhibit IP_3_R-mediated Ca2+ release **A-C.** Intracellular Ca^2+^ release in response to 0.5 μM ATP was followed in the mCherry-positive Fura-2-AM loaded COS-1 cells overexpressing 3xFLAG-empty vector (A), 3xFLAG-Bcl-2^wt^ (B) or 3xFLAG-Bcl-2^ΔC^ (C). The free extracellular Ca^2+^ was buffered by addition of 3 mM BAPTA. The obtained Fura-2 signals (F340/F380) were calibrated and representative traces are plotted as [Ca^2+^]. **D.** Quantitative analysis of the amplitude of the ATP-induced Ca^2+^ signals from 5 independent experiments (n > 110 cells) is plotted as mean ± S.E.M.

Next, we studied the role of the C-terminus in Bcl-2α's inhibitory function on IP_3_R-mediated Ca^2+^ signaling. Similar experiments were performed as described in Figure 4, comparing the effect of 3xFLAG-Bcl-2^wt^ *versus* 3xFLAG-Bcl-2^ΔC^ overexpression on ATP-induced IP_3_R-mediated Ca^2+^ release. In contrast to 3xFLAG-Bcl-2^wt^, which reduced Ca^2+^ release in response to ATP (0.5 μM), 3xFLAG-Bcl-2^ΔC^ was not able to suppress IP_3_R-mediated Ca^2+^ release (Figure 6). The 3xFLAG-proteins displayed similar expression levels (Supplementary Figure S1C), indicating that the failure of 3xFLAG-Bcl-2^ΔC^ to inhibit IP_3_Rs is not due to a lower expression level compared to the 3xFLAG-Bcl-2^wt^ protein. The ER Ca^2+^-store content was not changed in either of the conditions, pointing that the difference in ATP-induced Ca^2+^ rise is not due to a decreased ER Ca^2+^-store content (Supplementary Figure S1C).

### The TMD of Bcl-2α suppresses IICR in permeabilized cells and in single-channel recordings

**Figure 7:**
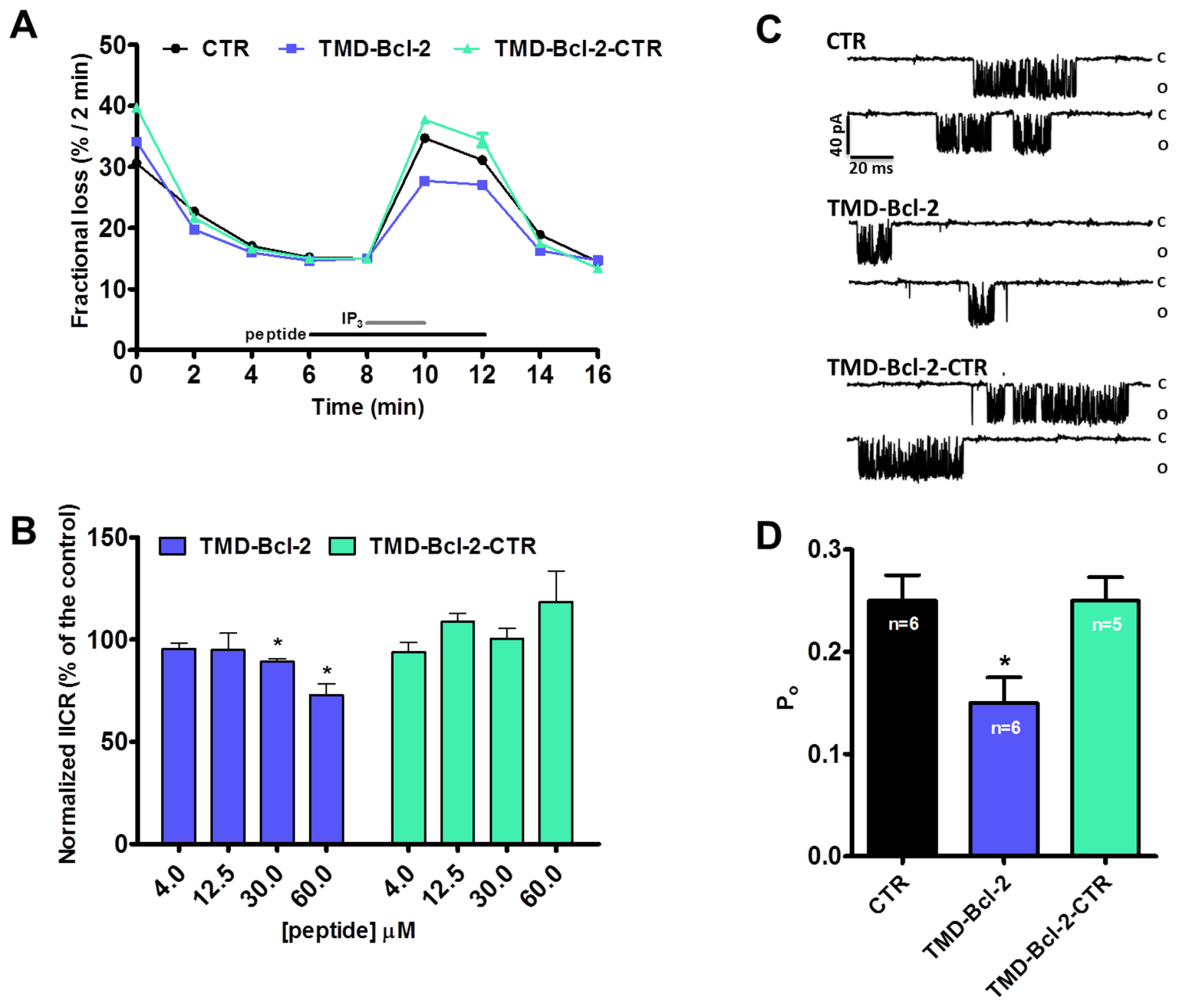
The TMD of Bcl-2 is sufficient to inhibit IP3Rs in permeabilized cell systems and single-channel recordings **A.** Typical experiment of unidirectional ^45^Ca^2+^ fluxes in permeabilized MEFs. Ca^2+^ release was induced by 3 μM IP_3_ (grey bar) in control condition or in presence of 60 μM peptides, TMD-Bcl-2 or TMD-Bcl-2-CTR (black bar). The results are plotted as fractional loss after 2 min of incubation with IP_3_ minus the fractional loss before the addition of IP_3_ (%/2 min) as a function of time. **B.** Quantification of the IICR from 5 independent experiments. The values of IICR measured as fractional loss were calculated as percentage of the IICR in control condition, which was set as 100%. **C.** Representative IP_3_R1 single-channel recordings from DT40 cells ectopically expressing IP_3_R1 evoked by 1 μM IP_3_ at 200 nM Ca^2+^ and 5 mM ATP, in control condition or in presence of the TMD-Bcl-2 or TMD-Bcl-2-CTR peptides. **D.** Histogram depicting the open probability (P_o_) ± SD for the IP_3_R1 under the previously described conditions. The total number of recordings for each condition is indicated within every bar.

We demonstrated that the TMD of Bcl-2α directly binds to the C-term Dom of IP_3_R and that Bcl-2^ΔC^ does not inhibit IP_3_R-mediated Ca^2+^ release. Next, we assessed whether the TMD of Bcl-2α by itself could affect the Ca^2+^-flux properties of the IP_3_R. Therefore, we performed unidirectional ^45^Ca^2+^ flux assays in saponin-permeabilized mouse embryonic fibroblasts (MEFs), in which non-mitochondrial Ca^2+^ stores were loaded with ^45^Ca^2+^. After loading, the unidirectional Ca^2+^ flux was measured in the presence of EGTA (1 mM) and in presence of Tg (4 μM). We quantified ^45^Ca^2+^ release triggered by IP_3_ (3 μM) in presence or absence of different concentrations of the synthetic peptides corresponding to the TMD of Bcl-2 or its mutated version. Peptides were applied 2 min before till 2 min after IP_3_ application. All conditions were matched to the vehicle control (DMSO). Data are plotted as the fractional loss (%/2 min) over time. These experiments indicated that high concentrations of TMD-Bcl-2 (30 μM and higher), but not of TMD-Bcl-2-CTR, suppress IICR without affecting ER Ca^2+^ level (Figure 7A, 7B). To further underpin these observations, IP_3_R1 single-channel recordings were performed using the nuclear-membrane patch-clamp technique on isolated nuclei obtained of triple-IP_3_R-knockout DT40 cells ectopically expressing IP_3_R1 [52]. This approach allows a direct measurement of the activity of the IP_3_R1 channel. IP_3_R1 single-channel activity was recorded in response to submaximal concentrations of IP_3_ (1 μM) in the presence of ATP (5 mM) and Ca^2+^ (200 nM). Figure 7C shows different representative traces of IP_3_R1 single-channel openings at a pipette holding potential of −100 mV in control conditions or in the presence of TMD-Bcl-2 or TMD-Bcl-2-CTR peptides, both at 60 μM final concentrations. TMD-Bcl-2 decreased the P_o_ of the IP_3_R1 channel from about 0.25 in the control conditions to about 0.15, whereas the TMD-Bcl-2-CTR peptide did not have any significant impact.

### Bcl-2α requires its TMD to suppress STS-induced apoptosis

**Figure 8:**
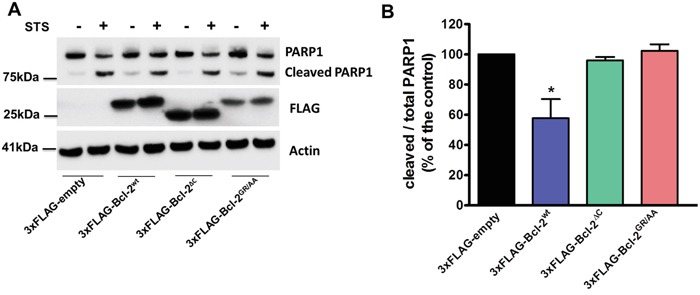
The TMD of Bcl-2 is required for STS-induced apoptosis **A.** Western-blot analysis for monitoring PARP1 cleavage upon staurosporine (STS) treatment (1 *μ*M for 6 h) in COS-1 cells overexpressing 3xFLAG-empty, 3xFLAG-Bcl-2^wt^, 3xFLAG-Bcl-2^GR/AA^ or 3xFLAG-Bcl-2^ΔC^. 7 μg of total cell lysate were loaded and the immunoblots were stained for PARP1, FLAG and Actin. **B.** Quantification of the ratio of the immunoreactive bands of cleaved over full-length PARP1 from 4 independent experiments, utilizing each time independently transfected COS-1 cells and freshly prepared lysates. The ratio of cleaved over full-length PARP1 obtained for control cells was set as 100% and the other ratios were normalized to this value. The data are plotted as ± S.E.M.

Finally, we studied the potency of overexpressed 3xFLAG-Bcl-2^wt^, 3xFLAG-Bcl-2^GR/AA^ and 3xFLAG-Bcl-2^ΔC^ to protect against STS, an apoptotic trigger that acts in part through Ca^2+^ signalling [53]. As a marker of apoptosis, we monitored the cleavage of poly-(ADP-ribose)-polymerase 1 (PARP1), which is a downstream target of activated Caspase-3. Compared to the control cells (transfected with an empty vector), the overexpression of 3xFLAG-Bcl-2^wt^ significantly reduced the levels of cleaved PARP1 upon STS treatment (1 μM, 6h). 3xFLAG-Bcl-2^GR/AA^ failed to prevent PARP1 cleavage, in line with its failure to bind Bax (Supplementary Figure S2). Despite the fact that 3xFLAG-Bcl-2^ΔC^ was equally efficient as the 3xFLAG-Bcl-2^wt^ to bind endogenous Bax (Supplementary Figure S2), it was much less efficient in preventing STS-induced apoptosis in COS-1 cells (Figure 8).

## DISCUSSION

**Figure 9:**
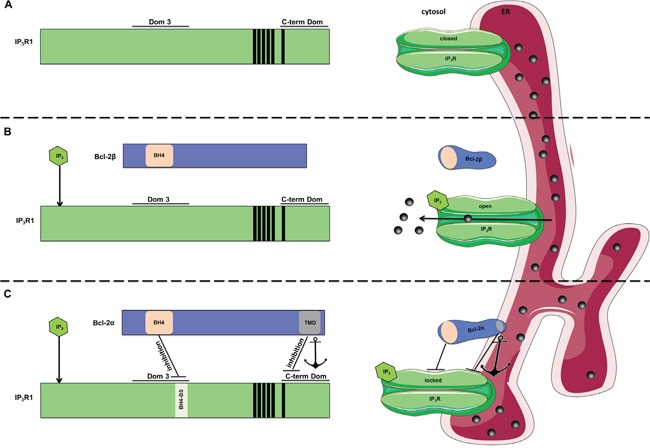
Model for inhibition of IP_3_Rs by Bcl-2 proteins The left side of the picture shows linear representation of the multi-domain interaction between Bcl-2 proteins and IP_3_R. On the right side these interactions are depicted within the ER membrane environment. **A.** Without IP_3_ present, IP_3_R is in closed conformation and no Ca^2+^ release occurs. **B.** Upon stimulation, IP_3_ binds to the N-terminal ligand-binding domain of IP_3_R and leads to change in the conformation of the channel from closed to open state. This results in IP_3_R-mediated Ca^2+^ release. Bcl-2β, which similarly to our Bcl-2^ΔC^, contains the BH4 domain, but lacks the TMD, might result in ineffective binding and regulation of the channel *in cellulo*. **C.** Efficient IP_3_R inhibition by Bcl-2α *in cellulo* requires multi-domain interaction between the two proteins, which involves binding of the BH4 domain of Bcl-2α to the Dom 3 of IP_3_R and binding between their C-termini. Here, we hypothesize that due to this multi-domain interaction, the IP_3_R is “locked” in a rigid conformation, leading to decreased Ca^2+^ release through the channel even in presence of IP_3_. We propose a model, in which the interaction between the TMD of Bcl-2α and the C-term Dom of IP_3_R can “concentrate” the BH4 domain in the proximity of the Dom 3 by serving as an anchoring mechanism (indicated with an anchor). This “concentration effect” could overcome the inherent low affinity of inhibition by the BH4 domain. In addition to its anchoring role, the TMD of Bcl-2α has an inhibitory effect by itself.

Here, we demonstrate that the efficient *in cellulo* suppression of IP_3_R activity by Bcl-2α protein requires the C-terminal region, containing the TMD, but not the hydrophobic cleft of Bcl-2α. Consistent with this finding, Bcl-2α lacking the TMD is less effective to protect cells against Ca^2+^-dependent pro-apoptotic stimuli like staurosporine. Since the TMD is present only in Bcl-2α, but not in Bcl-2β, our study is the first one that indicates a possible difference between the functional effects of the Bcl-2 isoforms on IP_3_R activity (Figure 9) and thus on Ca^2+^-dependent apoptosis. Furthermore, our data indicate that BH3-mimetic compounds like ABT-199, which selectively antagonizes Bcl-2, do not interfere with the functional regulation of IP_3_Rs by Bcl-2.

Using genetic and pharmacological approaches, we firmly ruled out a major role for the hydrophobic cleft of Bcl-2 in inhibiting IP_3_R function, despite the presence of previously suggested [54] or identified throughout this study putative BH3 motifs within the IP_3_R sequence (Figure 1B). This is in striking contrast to the regulation of IP_3_Rs by Bcl-Xl, very recently described to occur *via* a BH3-dependent mechanism, involving an interaction between the hydrophobic cleft of Bcl-Xl and two BH3 motifs in the C-term Dom of IP_3_R [54, 55]. Disruption of these interactions resulted in diminished cell viability. The authors speculated that similar BH3-dependent interactions might underlie the Bcl-2/IP_3_R complex [55]. Therefore, our work suggests that despite the similarities in their structure and function as inhibitors of the canonical Bax/Bak-dependent apoptosis, Bcl-2 and Bcl-Xl target and regulate IP_3_Rs by different mechanisms. The data reported here might concede another striking difference in addition to the documented selective function of Bcl-2 *versus* Bcl-Xl in regulating IP_3_Rs at the level of their BH4 domains [27]. Of note, selective BH3-mimetic molecules that could occupy the hydrophobic cleft of Bcl-2, but not that of Bcl-Xl, have been developed, indicating important differences in the molecular determinants contributing to the hydrophobic cleft of Bcl-2 and Bcl-Xl [50]. Hence, the BH3 motifs present in the IP_3_R might be suited for binding the hydrophobic cleft of Bcl-Xl, but not the one of Bcl-2. In addition, the hydrophobic cleft of Bcl-2 was recently excluded as a major contributor in the inhibition of another family of intracellular Ca^2+^-release channels, namely ryanodine receptors (RyRs) [45].

Previously we reported that the absence of the 6^th^ TMD of IP_3_R results in impaired Bcl-2 binding to the C-terminus of the channel [42]. Here, we demonstrate that the TMD of Bcl-2α is also required for this interaction, which likely occurs within the ER membrane. We propose that the TMD of Bcl-2α provides a concentration effect of Bcl-2 and its BH4 domain. This indicates that the membrane-dependent interaction between Bcl-2α and IP_3_R is critical for effective *in cellulo* inhibition of IP_3_R-mediated Ca^2+^ signaling and subsequent protection against Ca^2+^-dependent apoptosis. Our results also hint towards an unappreciated function of the TMD of Bcl-2α beyond its anchoring role for protein insertion into the membranes. Indeed, the TMD by itself is sufficient to inhibit IP_3_Rs as shown in unidirectional ^45^Ca^2+^ fluxes and single IP_3_R-channel recordings, correlating with previous findings that TMDs of other Bcl-2 family members play an important role in the protein functioning [56, 57]. However, it should be noted that even 60 μM TMD-Bcl-2 only partially inhibited IP_3_R-mediated Ca^2+^ release in permeabilized cells, indicating that also other Bcl-2 domains, particularly the BH4 domain, are required for efficient IP_3_R inhibition by Bcl-2α. Of note, the binding of Bcl-2α lacking its C-terminal region to the purified Dom 3 or to the full-length IP_3_Rs was not significantly disturbed, supporting the idea that the BH4 domain of Bcl-2 is sufficient for binding to the Dom 3 of IP_3_R and that this binding indeed occurs with relatively high affinity as documented *via* previous surface plasmon resonance analysis. We propose a model according to which the efficient IP_3_R inhibition relies on a complex multi-domain binding between Bcl-2α and IP_3_R, involving interactions between the BH4 domain of the former and the Dom 3 of the channel, and between the C-terminal regions of both proteins. We propose that the C-terminus of Bcl-2α (Figure 9C), but not the one of the Bcl-2β (Figure 9B), mediates the inhibitory effect of the BH4 domain by increasing its local concentration in the proximity of the Dom 3 of IP_3_R. In addition, the dual targeting of IP_3_Rs by Bcl-2α *via* its C-terminus and its BH4 domain might affect the conformational flexibility of the IP_3_R, by locking it in a rigid conformation and limiting the opening of the Ca^2+^-channel pore in response to IP_3_ (Figure 9). A particularly challenging aspect of our model is that, based on the most recent cryo-electron microscopy high-resolution structure of the IP_3_R1 [58], the 6^th^ TMD of the IP_3_R may not be readily available for interaction with other proteins. Yet, the published structure is in the absence of IP_3_ and thus likely represents the closed state. Hence, changes in the IP_3_R structure might arise in different IP_3_/Ca^2+^ conditions impacting the accessibility of the 6^th^ TMD of the IP_3_R to proteins like Bcl-2. *Vice versa*, it is also possible that the structure of IP_3_Rs loaded with Bcl-2 is different from the structure of IP_3_Rs in the absence of Bcl-2, thereby impacting the structural environment of the 6^th^ TMD of IP_3_R. Finally, we also would like to note that the molecular foundation for this model is mainly based on binding studies, using IP_3_R1-expression constructs and the electrophysiological analysis of IP_3_R1 channels. However, some of the cell models used for the functional analysis mainly express IP_3_R3 and IP_3_R1 isoforms [59]. As such, we anticipate that the important role of Bcl-2's TMD for efficient IP_3_R inhibition is not limited to IP_3_R1 channels, but further detailed molecular and functional work would be needed to firmly proof this. Of note, the BH4-domain-binding site present in IP_3_R1 is completely conserved in IP_3_R2 and IP_3_R3 [60], consistent with Bcl-2's ability to bind to the central domain of all three IP_3_R isoforms [27].

The importance of the multi-domain interaction between IP_3_Rs and Bcl-2 is underpinned by the fact that peptides antagonizing Bcl-2 at its BH4 domain (like Bcl-2/IP_3_ Receptor Disrupter-2; BIRD-2) are able to trigger pro-apoptotic Ca^2+^ signaling in a variety of cancer-cell models, including lymphoma, leukemia and lung cancer cells [35, 37, 61]. Thus, development of inhibitors targeting Bcl-2's TMD and interfering with the IP_3_R/Bcl-2 complex at the level of the TMD/C-term Dom interaction might further potentiate BH4-domain-antagonizing tools by helping to destabilize the Bcl-2/IP_3_R complex. Yet, given the hydrophobic nature of TMD/C-term Dom interactions, such small-molecule developments may prove to be very challenging.

We conclude that efficient IP_3_R regulation by Bcl-2α requires the TMD, a unique feature that discriminates Bcl-2α from Bcl-2β. Bcl-2α, *via* its TMD, likely “concentrates” its BH4 domain in the proximity of the central, modulatory domain of the IP_3_R, thereby facilitating its ability to efficiently suppress IP_3_R-mediated Ca^2+^ signaling and subsequent apoptosis.

## MATERIALS AND METHODS

### Peptides

The following peptides, obtained from Life Tein (Hillsborough, NJ, USA) with purity ≥ 85% were used: the peptide corresponding to the TMD of Bcl-2, Bcl-2-TMD: KTLLSLALVGACITLGAYLGHK (also used with biotin tag); the control peptide containing several mutations of hydrophobic residues, Bcl-2-TMD-CTR: KTRRSLADRGACRTRGAYDGHK (also used with biotin tag) and the peptide used to compete with the 3xFLAG tag, Anti-DYKDDDDK-tag peptide: MDYKDHDGDYKDHDIDYKDDDDK.

### Antibodies

The following antibodies were used: mouse monoclonal HRP-conjugated anti-FLAG M2 (1:1000; Sigma-Aldrich, Munich, Germany); mouse anti-FLAG M2 (1:1000, Sigma-Aldrich); mouse monoclonal HRP-conjugated anti-Bcl-2 (1:1000, Santa Cruz Biotechnology, Santa Cruz, CA, USA), mouse anti-GST (1:5000; Cell Signaling Technology, Danvers, Massachusetts, USA); mouse monoclonal anti-βActin (1:20 000, Sigma-Aldrich); rabbit anti-BAX (1:1000; Santa Cruz), rabbit anti-IP_3_R1 (1:1000; Rbt03 [62]); rabbit polyclonal anti-PARP-1 (1:1000, Alexis-Enzo Life Sciences, Farmingdale, NY, USA) as primary antibodies and secondary mouse and rabbit anti-IgG HRP conjugated antibodies (1:2500, Cell Signaling Technology).

### Plasmids, constructs and protein purification

pCMV24-3xFLAG-Myc constructs for expression of 3xFLAG–Bcl-2 and 3xFLAG-Bcl-2^GR/AA^ were obtained as previously described [45]. The 3xFLAG-Bcl-2^ΔC^ mutant, in which a stop codon was introduced at amino acid W214, was developed *via* PCR site-directed mutagenesis utilizing the following primers: Forward: 5′ GTTTGATTTCTCCTGACTGTCTCTGAAGACTC 3′ and Reverse: 5′ GAGTCTTCAGAGACAGTCAGGAGAAATCAAAC 3′.

BL21(DE3) *Escherichia coli* cells were transformed with pGEX-6p2 constructs containing cDNAs of parental GST, GST-Dom 3 of IP_3_R1 (a.a. 923-1581) or GST-C-term Dom of IP_3_R1 (a.a. 2512–2749), which were obtained as previously described [42]. The expressed parental GST or GST-fusion proteins were purified as previously described [42] and dialysed against standard phosphate-buffered saline (PBS) without Ca^2+^ and Mg^2+^ (Invitrogen, Merelbeke, Belgium) using Slide-A-Lyzer cassettes with a cut-off of 10 kDa (Thermo Fisher Scientific, Pittsburg, PA, USA). The concentration of the purified and dialysed proteins was determined using the Bradford assay (Sigma–Aldrich). Purity and quality were assessed after SDS–PAGE *via* total protein staining using the GelCode Blue Stain Reagent (Thermo Scientific, Rockford, IL, USA).

### Cell culture and transfections

All media and supplements used in this paper were purchased from Life Technologies (Ghent, Belgium) unless stated otherwise. COS-1 cells were cultured at 37°C, 10% CO_2_ in Dulbecco's Modified Eagle's medium (DMEM), containing 10% fetal calf serum (Sigma-Aldrich), 100 IU/ml penicillin, 100 μg/ml streptomycin, 2.5 μg/ml fungizone and 2 mM glutamax. MEF cells were cultured at 37°C in a 10% CO_2_ incubator in DMEM/Ham's F12 medium supplemented with 10% fetal calf serum, 3.8 mM L-glutamine, 85 IU/ml penicillin and 85 μg/ml streptomycin.

24 hours after seeding COS-1 cells were transiently transfected with empty p3xFLAG-Myc-CMV-24 (3xFLAG-empty) or with the same vector containing either Bcl-2^wt^ or the mutants Bcl-2^GR/AA^or Bcl-2^ΔC^. For co-IP and pull-down experiments JETPrime transfection reagent (Polyplus Transfections, Illkirch, France) was used according to the manufacturer's instructions. For single-cell cytosolic [Ca^2+^] measurements COS-1 cells were seeded in two-chamber slides. The same construct, in combination with a pcDNA 3.1(−) mCherry-encoding vector at a 3:1 ratio as selection marker, were introduced 24 hours after seeding using X-tremeGene HP DNA (Roche, Basel, Switzerland) as a transfection reagent according to the manufacturer's instructions.

### GST-pull down assays

48 hours after transfection COS-1 cells overexpressing 3xFLAG-Bcl-2^wt^, 3xFLAG-Bcl-2^GR/AA^ or 3xFLAG-Bcl-2^ΔC^ were harvested and lysed in a buffer containing 25 mM Tris-HCl (pH 7.5), 150 mM NaCl, 1.5 mM MgCl_2_, 0.5 mM DTT, 1% Triton X-100 and protease inhibitor cocktail tablets (Roche). After 30 min of incubation at 4°C the clear lysates were collected *via* centrifugation for 2 min at 10 000 rpm at 4°C. Parental GST, GST-Dom 3 or GST-C-term Dom (0.5 μM) were incubated together with 100 μg lysate in the lysing buffer (final volume 500 μl) at 4°C. After 1 hour the GST-proteins, used as bait, were immobilized on glutathione-Sepharose 4B beads (GE Healthcare, Diegem, Belgium) for 1.5 hour at 4°C. In order to study the effect of the BH3-mimetic compound, 3 μM ABT-199 (Active Biochem, Germany) or the vehicle control DMSO (Sigma-Aldrich, St Louis, MO) was added during the last hour of incubation. The beads were washed 5 times with the Triton X-100 buffer. The GST-complexes were eluted in 40 μl 2×LDS (Life Technologies) supplemented with 1:200 β-mercaptoethanol by boiling for 5 min at 95°C. Samples (10 μl) were analyzed *via* SDS-PAGE and the quantification was performed as previously described [63].

### Biotin-pull down assays

Equal amounts of the peptides (30 μg), biotin-TMD-Bcl-2 or biotin-TMD-Bcl-2-CTR, dissolved in 100% DMSO were incubated with 0.35 μM purified GST-C-term Dom of IP_3_R1 or parental GST (control) in interaction buffer (50 mM Tris-HCl, 200 mM NaCl, 0.1% NP-40, 1% BSA and protease inhibitor cocktail, pH 7.0) in a final volume of 400 μl. The incubation was performed over night at 4°C in a head-over-head rotator. The biotinylated peptides were immobilized on neutravidin agarose beads (Thermo Fisher Scientific, Pierce, Erembodegem, Belgium) and placed in a head-over-head rotator for 2 hours at 4°C. The beads were washed 7 times with the interaction buffer and the peptide-protein complexes were eluted by incubating the beads with 35 μl LDS supplemented with 1:200 β-mercaptoethanol for 3 min at 95°C. The eluates were collected after centrifuging at 2000 g for 1 min, using spin columns (Pierce) and 10 μl was analysed on NuPAGE 4–12% Bis/Tris SDS–polyacrylamide gels using MES/SDS-running buffer (Invitrogen).

### FLAG-co-immunoprecipitation assay

48 hours after transfection COS-1 cells overexpressing the control vector 3xFLAG-empty, 3xFLAG-Bcl-2^wt^, 3xFLAG-Bcl-Bcl-2^GR/AA^ or 3xFLAG-Bcl-2^ΔC^ were harvested and lysed in buffer containing 10 mM Hepes (pH 7.5), 0.25% NP-40, 142 mM KCl, 5 mM MgCl_2_, 2 mM EDTA, 2 mM EGTA and protease inhibitor cocktail tablets (Roche) as described for the GST-pull down assay. 100 μg of lysate was mixed with 30 μl anti-DYKDDDDK-tag conjugated resin (Biolegend, San Diego, CA) in the lysis buffer in total volume of 400 μl. The samples were incubated for 2.5 hours using a head-over-head rotor at 4°C. The beads were washed 2 times with washing buffer (50 mM Tris-HCl (pH 8.0), 150 mM NaCl, 1% NP-40, 0.5% sodium deoxycholate and 0.1% SDS) *via* centrifugation for 1 min at 3000g using spin columns. The FLAG-complexes were eluted by competitive incubation with the Anti-DYKDDDDK-tag peptide (10 μg, dissolved in 50mM Tris-HCl and 150mM NaCl) for 30 min at 15°C. To the resulting eluates, collected *via* centrifugation for 1 min at 500g, 25 μl LDS supplemented with 1:200 β-mercaptoethanol was added. 15 μl of each sample was analysed on NuPAGE 4–12% Bis/Tris SDS–polyacrylamide gels using MES/SDS-running buffer.

### Single-cell cytosolic Ca^2+^ imaging

Fura-2-AM [Ca^2+^] measurements in COS-1 cells were performed as previously described [27]. The effect of ABT-199 was studied by incubating the cells with 3 μM of the compound or DMSO for 1 hour (during the incubation procedure with Fura-2 AM). BAPTA (3 mM) was added for 1 minute prior to the stimulation with ATP or Tg to chelate all free extracellular Ca^2+^. Cytosolic Ca^2+^ rises in response to 0.5 μM ATP or 2.5 μM Tg were measured in mCherry-positive (excitation 546 nm, emission 610 nm) and Fura-2-loaded cells. Intracellular cytoplasmic Ca^2+^ concentrations were calculated as previously described [27].

### Unidirectional ^45^Ca^2+^-flux assay

The unidirectional ^45^Ca^2+^-flux experiments were performed in permeabilized MEFs as previously described [27]. IICR was triggered during the unidirectional ^45^Ca^2+^-efflux phase by the addition of 3 μM IP_3_ for 2 min. Peptides were added 2 min before IP_3_ till 2 min after IP_3_. IICR was plotted as fractional loss, representing the amount of Ca^2+^ leaving the store in a 2-min time period divided by the total store Ca^2+^ content at that time point as a function of time [64].

### Preparation of GUVs and electrophysiological analysis

Isolation of the ER-containing membrane fractions from control and Bcl-2-expressing WEHI7.2 cells and preparation of the GUVs were carried out as described previously [65]. GUVs were prepared from the 1:5 mixtures of the ER-containing fraction with 10:1 diphytanoylphosphatidylcholine/cholesterol lipid combination (5 mM). The Patch-clamp experiments were carried out using Axopatch 200B amplifier and pClamp 10.0 software (Molecular Devices, Union City, CA) for data acquisition and analysis. Patch pipettes were fabricated from borosilicate glass capillaries (World Precision Instr., Inc., Sarasota, FL) on a horizontal puller (Sutter Instruments Co., Novato, CA) and had a resistance in the range of 7-10 MΩ. Prepared vesicles were immersed in a bath solution containing 150 mM KCl, 10 mM Hepes, 5 mM glucose, pH 7.2. Patch pipettes were filled with the same solution.

### Isolation of nuclei and electrophysiological analysis

Isolated DT40 nuclei were prepared by homogenization as previously described [52]. A 3 *μ*l aliquot of nuclear suspension was placed in 3 ml of bath solution which contained 140 mM KCl, 10 mM Hepes, 500 *μ*M BAPTA and 246 nM free Ca^2+^, pH 7.1. Nuclei were allowed to adhere to a plastic culture dish for 10 min prior to patching. Single IP_3_R channel potassium currents (*i*k) were measured in the on-nucleus patch clamp configuration using pCLAMP 9 and an Axopatch 200B amplifier (Molecular Devices, Sunnydale, CA, USA) as previously described [66]. Pipette solution contained 140 mM KCl, 10 mM Hepes, 1 μM IP_3_, 5 mM ATP, and 200 nM free Ca^2+^ as well as 60 μM TMD-Bcl-2 or TMD-Bcl-2-CTR peptides. Traces were consecutive 3 s sweeps recorded at −100 mV, sampled at 20 kHz and filtered at 5 kHz. A minimum of 15 s of recordings were considered for data analyses. Pipette resistances were typically 20 MΩ and seal resistances were *>*5 GΩ. Single channel openings were detected by half-threshold crossing criteria using the event detection protocol in Clampfit 9. We assumed that the number of channels in any particular nuclear patch is represented by the maximum number of discrete stacked events observed during the experiment. Only patches with one apparent channel were considered for analyses.

### Apoptosis induction and analysis

COS-1 cells were transiently transfected with 3xFLAG-vectors and treated with 1 μM STS (Sigma-Aldrich). After 6h the cells were harvested and lysed in a buffer containing 25 mM Hepes (pH 7.5), 1% Triton X-100, 10% glycerol, 0.3 M NaCl, 1.5 mM MgCl_2_, 1 mM DTT, 2 mM EDTA, 2 mM EGTA and protease inhibitor cocktail tablets (Roche). Apoptosis progression was monitored *via* Western-blotting analysis of PARP1 cleavage in 10 μg total lysate.

### Sequence alignment and secondary-structure predictions

The amino acid sequences of the BH3 domains of Bcl-2 proteins and the Dom 3 of IP_3_R were taken from the National Center for Biotechnological Information's nonredundant database. The I-TASSER v 2.1 webserver [67, 68] was used to predict the secondary structure of the BH3-like motif identified in the Dom 3 of IP_3_R1. I-TASSER builds protein models using iterative assembling procedures and multiple threading alignments from template structures libraries. An estimate of accuracy of the predictions is given by the confidence score. The most accurate I-TASSER model was downloaded as PDB file and imported in PyMOL, a molecular graphic software (http://www.pymol.org).

### Statistical analysis

Two-tailed unpaired Student's *t*-tests were performed when two conditions were compared. When comparing three or more conditions a repeated measure ANOVA with Bonferroni post test was performed. * indicates significantly different results with *p* < 0.05.

## SUPPLEMENTARY FIGURES


