# Supplementary material for: The trans-membrane domain of Bcl-2α, but not its hydrophobic cleft, is a critical determinant for efficient IP3 receptor inhibition
Source: Oncotarget. 2016 Aug 2;7(34):55704–20. doi: 10.18632/oncotarget.11005 (PMC5342447; doi:10.18632/oncotarget.11005)
Supplement: Supplementary file 1 [file oncotarget-07-55704-s001.pdf]

The trans-membrane domain of Bcl-2α, but not its hydrophobic cleft, is a critical determinant for efficient IP_3 receptor inhibition

SUPPLEMENTARY FIGURES

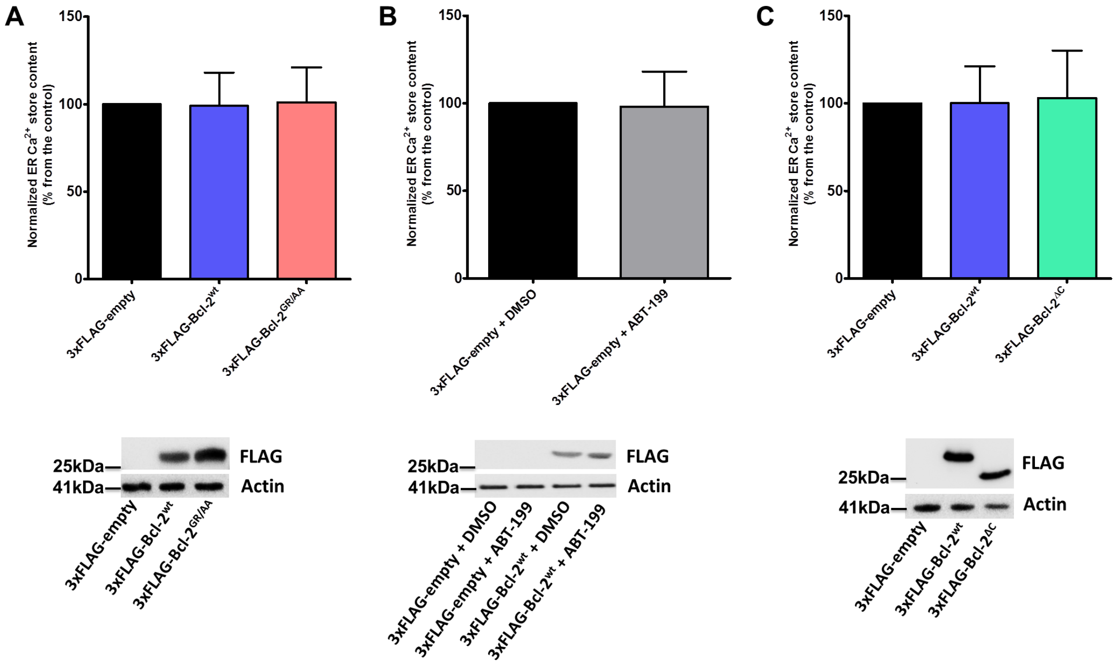

Supplementary Figure S1: Overexpression of 3xFLAG-Bcl-2^wt^ or its mutants and presence of ABT-199 do not change the ER Ca^{2+} content. A-C. Top: Quantitative analysis of the area under the curve of Tg-induced Ca^{2+} signals in COS-1 cells overexpressing 3xFLAG-empty vector, 3xFLAG-Bcl-2^{wt} or 3xFLAG-Bcl-$2^{GR/AA}$ (A), 3xFLAG-empty vector in absence or presence of 3μM ABT-199 (B) and 3xFLAG-empty vector, 3xFLAG-Bcl-2^{wt} or 3xFLAG-Bcl-$2^{\Delta C}$ (C). The results are plotted as percentage of the control 3xFLAG-empty vector. A-C. Bottom: Representative Western blot analysis of the expression levels of the 3xFLAG proteins in the COS-1 cells used in the Ca^{2+} measurements. The immunoreactive bands were stained with the respective antibody against FLAG or actin.

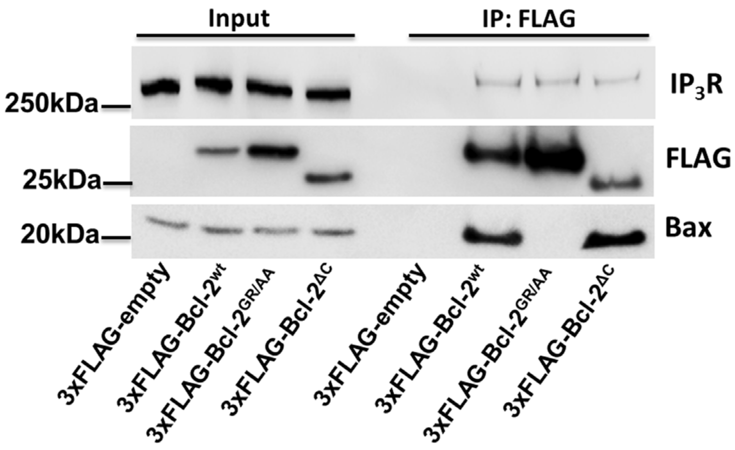

Supplementary Figure S2: Bcl-2$^{GR/AA}$ remains capable of binding to the full-size IP$_3$Rs, but not to Bax, while Bcl-2$^{\Delta C}$ binds to both proteins. Representative FLAG-coIP experiment for assessing the Bcl-2/IP$_3$R and Bcl-2/Bax interactions in COS-1 cells overexpressing 3xFLAG-Bcl-2^{wt}, 3xFLAG-Bcl-2$^{GR/AA}$ or 3xFLAG-Bcl-2$^{\Delta C}$ is shown. The samples were analyzed *via* Western blot and stained with the respective antibody against FLAG, IP$_3$R1 or Bax. 0.1 µg and 6 µg of total COS-1 lysates were used as input for the 3xFLAG-proteins and IP$_3$R or Bax respectively. The experiments were performed 3 times utilizing each time independently transfected cells and freshly prepared lysates.
